# Reactive Oxygen Species Regulate Endoplasmic Reticulum Stress and ER-Mitochondrial Ca^2+^ Crosstalk to Promote Programmed Necrosis of Rat Nucleus Pulposus Cells under Compression

**DOI:** 10.1155/2021/8810698

**Published:** 2021-03-16

**Authors:** Hui Lin, Yizhong Peng, Jinye Li, Zhe Wang, Sheng Chen, Xiangcheng Qing, Feifei Pu, Ming Lei, Zengwu Shao

**Affiliations:** ^1^Department of Orthopedics, Union Hospital, Tongji Medical College, Huazhong University of Science and Technology, Wuhan 430022, China; ^2^Department of Gastroenterology, Union Hospital, Tongji Medical College, Huazhong University of Science and Technology, Wuhan 430022, China

## Abstract

Programmed necrosis of nucleus pulposus (NP) cells caused by excessive compression is a crucial factor in the etiopathogenesis of intervertebral disc degeneration (IVDD). The endoplasmic reticulum (ER) and mitochondria are crucial regulators of the cell death signaling pathway, and their involvement in IVDD has been reported. However, the specific role of ER stress (ERS) and ER-mitochondria interaction in compression-induced programmed necrosis of NP cells remains unknown. Our studies revealed that compression enhanced ERS and the association between ER and mitochondria in NP cells. Suppression of ERS via 4-phenylbutyrate (4-PBA) or ER-mitochondrial Ca^2+^ crosstalk by inhibiting the inositol 1,4,5-trisphosphate receptor, glucose-regulated protein 75, voltage-dependent anion-selective channel 1 complex (IP_3_R–GRP75–VDAC1 complex) protected NP cells against programmed necrosis related to the poly(ADP-ribose) polymerase (PARP) apoptosis-inducing factor (AIF) pathway. Moreover, excessive reactive oxygen species are critical activators of ERS, leading to mitochondrial Ca^2+^ accumulation and consequent programmed necrosis. These data indicate that ERS and ER-mitochondrial Ca^2+^ crosstalk may be potential therapeutic targets for the treatment of IVDD-associated disorders. These findings provide new insights into the molecular mechanisms underlying IVDD and may provide novel therapeutic targets.

## 1. Introduction

As the most common musculoskeletal disorder in outpatients, low back pain (LBP) causes huge economic losses in the global health system [1]. In the United States, this acute illness results in a loss of more than $100 billion in annual health care costs [2]. Intervertebral disc degeneration (IVDD) is the most common cause of LBP [3]. Excessive mechanical loads play a significant role in the etiology of IVDD [4]. Unphysiological loading exacerbates disc degeneration by accelerating disc cell death, leading to progressive loss of extracellular matrix and disc bioactivity [5]. However, the mechanisms underlying mechanical load-induced nucleus pulposus (NP) cell death have not been completely elucidated. Therefore, it is paramount to understand the molecular mechanisms of NP cell death under excessive mechanical loading conditions to identify effective therapies for IVDD treatment.

Mounting evidences indicate that programmed necrosis plays a greater role in the development of IVDD than the other two programmed cell death, apoptosis and autophagic cell death [6]. The most intuitive evidence is that necrotic cells in degenerated intervertebral discs account for more than 80% of the total [7]. In our previous study, NP cells showed mainly necrotic morphology changes under harmful stimuli, and inhibition of programmed necrosis by Nec-1 evidently retarded NP cell death [8]. Inhibition of apoptosis did not effectively relieve compression-induced cell death [9]. Therefore, mechanical load-induced NP cell death is mainly attributed to programmed necrosis. However, the underlying molecular mechanisms remain unclear.

The endoplasmic reticulum (ER) is the primary location for synthesis and maturation of proteins in response to cellular stimuli [10]. Additionally, ER is an essential location for intracellular Ca^2+^ store that plays a crucial role in signal transduction [11]. Under severe or prolonged ER dysfunction, ER stress (ERS) triggers cell death by the release of Ca^2+^ and subsequent triggering of a series of signal transduction pathways. Increasing evidence supports the involvement of ERS-initiated cell death in IVDD [12, 13]. Zhao et al. found that disc degeneration was concomitant with increased cell death and upregulation of ERS markers, caspase-12 and the 78 kDa glucose-regulated protein (GRP78) [14]. Wang et al. reported that IVDD at the mild stage showed a strong upregulation of ERS markers, including GRP78, growth arrest- and DNA damage-inducible gene 153, and caspase-12 [15]. However, the specific role of ERS in compression-induced programmed necrosis of NP cells remains unclear, and it is crucial to understand the underlying mechanisms for developing alternative treatment options for IVDD.

Mitochondrial dysfunction is a common pathophysiological change that occurs under disc overloading and contributes to IVDD [16]. Recent studies have shown that the mitochondria and ER interact physically and functionally to regulate their functions [17]. However, it is unclear how the interaction between ER and mitochondria is involved in compression-induced programmed necrosis of NP cells. Previous studies have confirmed that the ER couples with the mitochondria and an inositol 1,4,5-trisphosphate receptor (IP_3_R), glucose-regulated protein 75 (GRP75), voltage-dependent anion-selective channel 1 (VDAC1) complex (IP_3_R–GRP75–VDAC1 complex) is present at the ER-mitochondria interface, which is considered critical determinants of cell survival or death by exerting intracellular Ca^2+^ efflux into the mitochondria [18]. However, the involvement of the IP_3_R–GRP75–VDAC1 complex in compression-induced NP cell death has not been clarified.

In the current study, we demonstrated that ERS plays a critical role in compression-induced programmed necrosis of NP cells. Mechanical loading enhanced the association between ER and mitochondria, which in turn stimulated Ca^2+^ efflux to mitochondria through the IP_3_R–GRP75–VDAC1 complex, resulting in poly(ADP-ribose) polymerase (PARP)/apoptosis-inducing factor (AIF) pathway-related cell programmed necrosis. This study also clarified the mechanism by which ERS contributes to compression-induced programmed necrosis of NP cells. Our findings may provide a molecular basis for the inhibition of ERS or aberrant Ca^2+^ translocation in the treatment of IVDD.

## 2. Materials and Methods

### 2.1. Cell Culture

All animal experiments and protocols were approved by the animal experimentation ethics committee at the Huazhong University of Science and Technology. After euthanasia by intraperitoneal injection of pentobarbital sodium (100 mg/kg), NP tissues (L1-L7) of Sprague-Dawley rats (male, weight 250–300 g) were harvested using forceps, with a horizontal incision on the annulus fibrosis. NP tissues were then digested for 15 min in 0.25% type II collagenase (Gibco, USA) at 37°C. The digested NP tissue was cultured in Dulbecco's modified Eagle medium/Ham's F-12 (Gibco, USA) supplemented with 10% fetal bovine serum (Gibco, USA) and 1% penicillin/streptomycin (Sigma, USA) at 37°C. Cells were passaged till they reached 80–90% confluency. We used the second-generation cells in the following experiments.

### 2.2. Excessive Compression Loading on Rat NP Cells

A pressure apparatus was used to create excessive mechanical loading. Cells were seeded in 6-well plates (5.0 × 10^4^ per well) or 96-well plates (0.5 × 10^4^ per well) and cultured for 2 days before starting the experiments. As described previously [6], after pretreatment with 4-phenylbutyrate (4-PBA; Sigma, USA), dantrolene (DAN; Selleck, USA), xestospongin C (XeC; APExBIO, USA), ruthenium red (RR; Sigma, USA), or N-acetylcysteine (NAC; Sigma, USA) for 1 h, NP cells were placed in a pressure apparatus under the compression of 1 MPa. The apparatus was then sealed carefully. Two conduits were opened to transmit oxygen and carbon dioxide, ensuring 20% O_2_ and 5% CO_2_. A compressor was applied to create an atmosphere of 1.0 MPa. Cells were cultured under compression for 0, 12, 24, and 36 h. All cells were cultured for the same time span; only the duration of compression and the applied treatments varied.

### 2.3. Transmission Electron Microscopy (TEM)

TEM was used to examine changes in the ultrastructure of the cells [6]. Briefly, cells were trypsinized, collected, and washed twice with phosphate-buffered saline (PBS). Cell pellets were then created by centrifugation at 1500 × g for 20 min. Next, the cell pellets were treated with 2.5% glutaraldehyde for 2 h and then with 1% osmium tetroxide for 2 h. An ascending ethanol series was applied to dehydrate the pellets, and the cells were embedded in epon 812 (SPI, USA). Finally, after staining, sections were observed using TEM (FEI Company, Holland).

### 2.4. Propidium Iodide Staining

The cells were stained with 4 mg/mL Hoechst 33258 (Beyotime, China) for 5 min, then washed three times with PBS. The cells were covered with propidium iodide (PI; Beyotime, China), diluted with the corresponding buffer to 5 mg/mL. After incubation for 5 min, the NP cells were observed using a fluorescence microscope (Olympus IX71, Japan).

### 2.5. Flow Cytometric Analysis of PI Uptake

After treatment, NP cells were collected by trypsinization. Next, the NP cells were resuspended in 5 mg/mL PI for 15 min. The PI-positive cells were then determined by flow cytometry (Becton Dickinson, USA).

### 2.6. Lactate Dehydrogenase Leakage Assay

NP cells seeded in 96-well plates were exposed to a mechanical load in a pressure apparatus under the compression of 1 MPa. At the end of treatment, the lactate dehydrogenase (LDH) leakage assay kit (Beyotime, China) was used to detect NP cell necrosis, according to the manufacturer's instructions.

### 2.7. Immunohistochemistry

Experimental protocols involving human tissues were carried out following the Code of Ethics of the World Medical Association (Declaration of Helsinki) for experiments involving humans and approved by the Medical Ethics Committee of Tongji Medical College of Huazhong University of Science and Technology. Informed consent was obtained from all the participating subjects. Three patients with IVDD and three patients with scoliosis but without IVDD were chosen for immunohistochemical detection. In brief, after fixation with 4% paraformaldehyde for 24 h, human NP specimens were embedded in paraffin and sectioned (4 *μ*m thick). The sections were deparaffinized with xylene and rehydrated with graded ethanol to distilled water. Endogenous peroxidase activity was eliminated with 3% H_2_O_2_ for 15 min. Afterwards, the sections were washed three times with TBS, incubated with trypsin for 30 min, and then blocked with 10% goat serum (Solarbio, China) for 15 min. Next, the sections were incubated with antibodies against the C/EBP homologous protein (CHOP, 1 : 100; Abcam, UK) and GRP78 (1 : 250; Abcam, UK) overnight at 4°C, followed by incubation with secondary antibody (1 : 5000, Beyotime, China) and counterstained with hematoxylin.

### 2.8. Measurement of Mitochondrial Ca^2+^ ([Ca^2+^]_m_) Levels

Mitochondrial Ca^2+^ was detected using the Ca^2+^ probe Rhod-2 AM (MCE, USA). Cells were incubated with 10 *μ*M Rhod-2 AM for 2 h in the dark, washed twice with PBS, and then resuspended in PBS. Fluorescence was determined by flow cytometry, and fluorescence photomicrographs were taken using a fluorescence microscope.

### 2.9. Immunofluorescence Staining

The samples were fixed with 4% paraformaldehyde and permeabilized with 0.2% Triton X-100. Next, samples were incubated with primary antibodies against IP_3_R1 (1 : 500; Invitrogen, UK), VDAC1 (1 : 500; Abcam, UK), AIF (1 : 500; Abcam, UK), CHOP (1 : 500; Abcam, UK), or GRP78 (1 : 500; Abcam, UK) for 1 h at room temperature. Alexa Fluor-conjugated secondary antibodies (1 : 100; Proteintech, China) were added to highlight the primary antibodies. After one hour, the cells were visualized under a laser scanning confocal microscope (LSM; Olympus, Japan).

### 2.10. Coimmunoprecipitation

Cells were lysed in lysis buffer (Beyotime, China) mixed with 1% protease inhibitor (Beyotime, China) to extract total protein. Lysates were centrifuged at 13,000 rpm for 10 min at 4°C to pelletize the cell debris. Protein concentration was measured using the BCA protein assay kit (Beyotime, China). Coimmunoprecipitation (co-IP) was performed using protein A-coated sepharose beads following the manufacturer's instructions. Briefly, freshly prepared precleared lysates were incubated overnight at 4°C with anti-IP_3_R1 (1 : 500, Invitrogen, UK) or anti-VDAC1 (1 : 1000, Abcam, UK) antibodies. Protein A beads were added and incubated for 2 h at 4°C, with rocking. The proteins pulled down by anti-IP_3_R1 or anti-VDAC1 antibodies were analyzed by western blotting [19].

### 2.11. Measurement of Mitochondrial Membrane Potential

The JC-1 Mitochondrial Membrane Potential Assay Kit (Beyotime, China) was used to visualize mitochondrial membrane potential (MMP). After treatment, NP cells were washed with PBS and treated with 5 *μ*M JC-1 dye for 30 min. MMP loss was quantified by flow cytometry. A fluorescence microscope (Olympus IX71, Japan) was used to capture fluorescent signals.

### 2.12. Cellular ATP Assay

Briefly, NP cells were harvested and homogenized in a glass homogenizer for 3 min. The samples were then detected using an ATP assay kit (Nanjing Jiancheng, China) according to the manufacturer's instructions and analyzed using a spectrophotometer (Thermo Fisher Scientific, USA).

### 2.13. Measurement of Reactive Oxygen Species Production

Intracellular reactive oxygen species (ROS) levels were measured using the H_2_-DCFHDA assay (Beyotime, China). After treatment, NP cells were washed, then incubated with 10 *μ*M H_2_-DCFHDA for 30 min. Fluorescence was determined by flow cytometry, and cells were visualized under an LSM.

### 2.14. Detection of 8-Hydroxy-2′-deoxyguanosine (8-OHdG)

The detection of 8-OHdG (Cusabio, China) was performed as previously described [20]. DNA from NP cells was extracted using a DNA Extractor WB kit. The levels of 8-OHdG were detected using an ELISA assay kit according to the manufacturer's protocol.

### 2.15. Transfection of Small Interfering RNA

Small interfering RNAs (siRNAs) against rat GRP75 and VDAC1 were purchased from Biomics (Biomics Biotechnologies Co. Ltd, China). The sequence of GRP75-siRNA was as follows: 5′-GGAUAUAUAUCUAGAAUAAGAdTdT-3′, 5′-UUAUUCUAGAUAUAUAUCCUAdTdT-3′; the sequence of VDAC1-siRNA was as follows: 5′-GGAUACACUCAGACUCUAAAGdTdT-3′, 5′-UUAGAGUCUGAGUGUAUCCUAdTdT-3′. Cells were transfected with siRNA oligonucleotides using Lipofectamine RNAiMAX (Invitrogen, USA). Next, the culture medium was replaced with a complete culture medium.

### 2.16. Reverse-Transcription-Quantitative Polymerase Chain Reaction (RT-qPCR)

A TRIzol reagent (Invitrogen, USA) was used to extract total RNA from cells according to the manufacturer's instructions. RNA was transcribed to generate complementary DNA (cDNA), using HiScript II Q RT SuperMix (Vazyme, China). The primer sequences were as follows: CHOP: 5′-TTCTCTGGCTTGGCTGACTG-3′, 5′-TGTTTCCGTTTCCTGGTTCTCC-3′; GRP78: 5′-GGAGGAGGACAAGAAGGAGGAC-3′, 5′-CAGGAGTGAAGGCGACATAGG-3′; PERK: 5′-TGGATGATGTGGTCAAGGTTGG-3′, 5′-TCCTGTGTGTCTGGCATAAGC-3′; and GAPDH: 5′-CGCTAACATCAAATGGGGTG-3′, 5′-TTGCTGACAATCTTGAGGGAG-3′. Quantitative PCR (qPCR) assays were performed using SYBR Green Real-time PCR Master Mix (Toyobo, Japan) in a StepOnePlus Real-Time PCR System (Applied Biosystems, Canada). The data were analyzed using 2^-*ΔΔ*CT^ calculations.

### 2.17. Western Blot (WB) Analysis

Cells were lysed in lysis buffer (Beyotime, China) mixed with 1% protease inhibitors (Beyotime, China) to extract total protein. Mitochondrial fractions were extracted using a Cell Mitochondria Isolation Kit (Beyotime, China). The cell medium was centrifuged (800 × g, 5 min), and the supernatant was filtered for the detection of high mobility group box 1 protein (HMGB1), as described previously [17]. Protein concentrations were determined using a bicinchoninic acid (BCA) protein assay kit (Beyotime, China). Total cell lysates were separated by SDS polyacrylamide gel electrophoresis (SDS-PAGE) and transferred to polyvinylidene fluoride membranes (Amersham Biosciences, USA). Blots were probed with the primary antibodies against phosphorylated PKR-like ER kinase (p-PERK, 1 : 1000; CST, USA), GRP78 (1 : 1000; Abcam, UK), CHOP (1 : 1000; Abcam, UK), HMGB1 (1 : 10000; Abcam, UK), PARP (1 : 500; Abcam, UK), VDAC1 (1 : 1000; Abcam, UK), or GAPDH (1 : 1000; Tianjin Sun gene Biotech Co, China) and incubated (with rocking) at 4°C overnight. Membranes were probed with secondary antibodies and visualized using an enhanced chemiluminescence reagent (ECL, Amersham Biosciences, Piscataway, NJ, USA).

### 2.18. Statistical Analysis

Each experiment was performed for at least three biological replicates and presented as the mean ± standard deviation (SD) and analyzed using SPSS 19. Student's *t*-test was used to analyze the differences between the two groups. Multiple sets of data were analyzed by one-way repeated measures analysis of variance (ANOVA), followed by least significant difference (LSD). *P* < 0.05 was considered statistically significant.

## 3. Results

### 3.1. ERS Is Involved in Compression-Induced Programmed Necrosis of NP Cells

**Figure 1:**
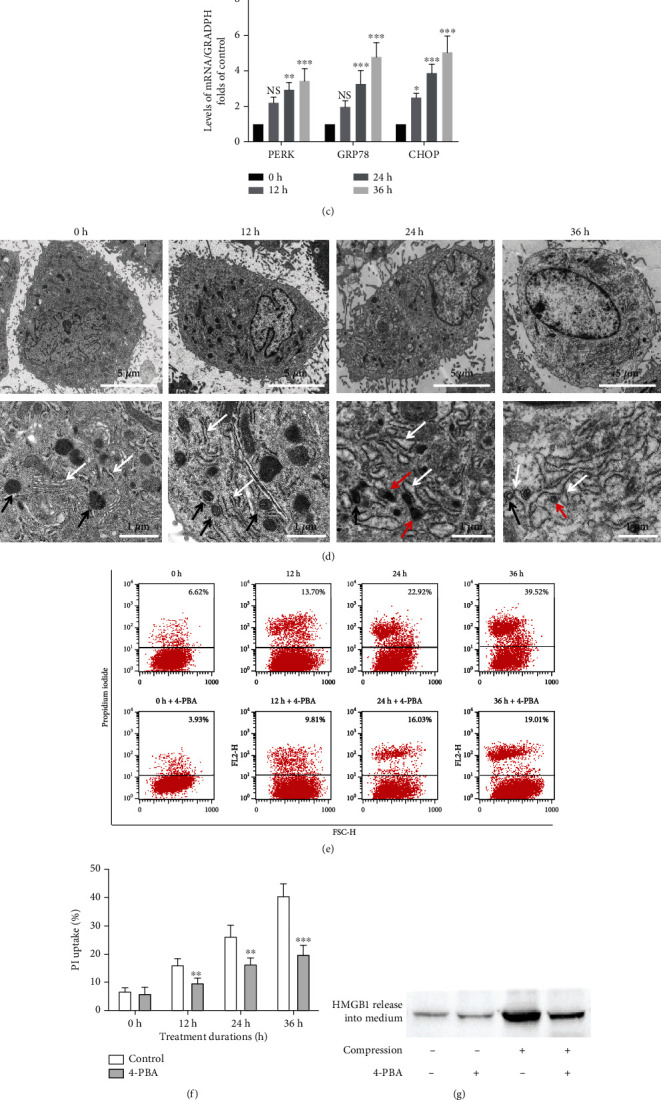
ERS plays a critical role in compression-induced programmed necrosis of NP cells. (a) Representative fluorescent images of GRP78 and CHOP staining in normal and degenerated discs detected by immunohistochemistry. (b) Representative western blot graph of the levels of p-PERK, GRP78, and CHOP protein expression in NP cells exposed to mechanical load for 0, 12, 24, and 36 h. (c) The mRNA levels of PERK, GRP78, and CHOP detected by RT-qPCR in NP cells under compression conditions. NS means no significant difference. The values are expressed as the mean ± SD from three biological replicates (^∗^*P* < 0.05, ^∗∗^*P* < 0.01, and ^∗∗∗^*P* < 0.001 vs. control, ANOVA/LSD). (d) Ultrastructural observations of rat NP cell morphology by TEM (ER: white arrowheads; mitochondria: black arrowheads; mitochondria interacting with ER: red arrowheads). (e, f) Representative dot plots and quantitative analysis of PI uptake in NP cells. Cells were pretreated with 200 *μ*M 4-PBA for 1 h and then subjected to compression for 0, 12, 24, and 36 h. The values are expressed as the mean ± SD from three biological replicates (^∗∗^*P* < 0.01 and ^∗∗∗^*P* < 0.001 vs. control, Student's *t*-test). (g) Representative western blot graphs of the levels of HMGB1 content in the media. Cells were pretreated with 200 *μ*M 4-PBA for 1 h and then subjected to compression for 36 h. (h) Histogram for statistical analysis of the LDH leakage in compression-treated NP cells. NP cells were treated as in (g). The values are expressed as the mean ± SD from three biological replicates (^∗∗∗^*P* < 0.001 vs. control, ^##^*P* < 0.01 vs. compression alone, ANOVA/LSD).

Increasing evidence supports the involvement of the ERS-initiated cell death pathway in IVDD [12, 13]. However, the precise role of ERS in compression-related NP death has not been fully elucidated. To confirm the existence of ERS in the pathogenesis of IVDD, we compared the levels of ERS markers in normal and degenerative discs. As seen in Figure 1(a) and Figure S1, the degenerated discs exhibited increased expression of GRP78 and CHOP compared to the nondegenerated discs. Next, we evaluated whether compression induced ERS in vitro. We examined the changes in ERS-related protein (CHOP, GRP78, and p-PERK) expression in NP cells under compression conditions. After treatment with compression for 0, 12, 24, and 36 h, the expression levels of CHOP, GRP78, and p-PERK increased in a time-dependent manner (Figure 1(b)). Consistently, compression significantly increased the mRNA levels of CHOP, GRP78, and PERK (Figure 1(c)). Subsequently, ER swelling was observed by transmission electron microscopy. NP cells without compression showed normal morphology with intact ER, while compression stimulation caused extensive expansion and deformation of ER, as indicated by white arrowheads. The borders between some mitochondria and ER were unclear at 24 h and 36 h, which indicated an interaction between ER and mitochondria; these mitochondria are highlighted by red arrowheads (Figure 1(d)).

To further investigate the effects of ERS on programmed necrosis caused by compression, NP cells were incubated with 4-PBA before compression treatment, and programmed necrosis of cells was examined. The elevation of CHOP, GRP78, and p-PERK levels was notably ameliorated by 4-PBA, suggesting that 4-PBA was sufficient to inhibit ERS in NP cells (Figure S2 (a)). Meanwhile, we found by flow cytometry (*P* < 0.01) and PI staining (Figures 1(e) and 1(f); Figure S2 (b)) that 4-PBA significantly attenuated compression-induced programmed necrosis in NP cells. In addition, the detection of HMGB1 expression and LDH release also showed that 4-PBA strongly inhibited programmed necrosis of NP cells induced by the mechanical load (*P* < 0.01; Figures 1(g) and 1(h)). Our data indicated that ERS was activated under prolonged compression, while its inhibition significantly alleviated compression-induced programmed necrosis in NP cells.

### 3.2. Compression-Induced Ca^2+^ Mitochondrial Translocation from the ER

**Figure 2:**
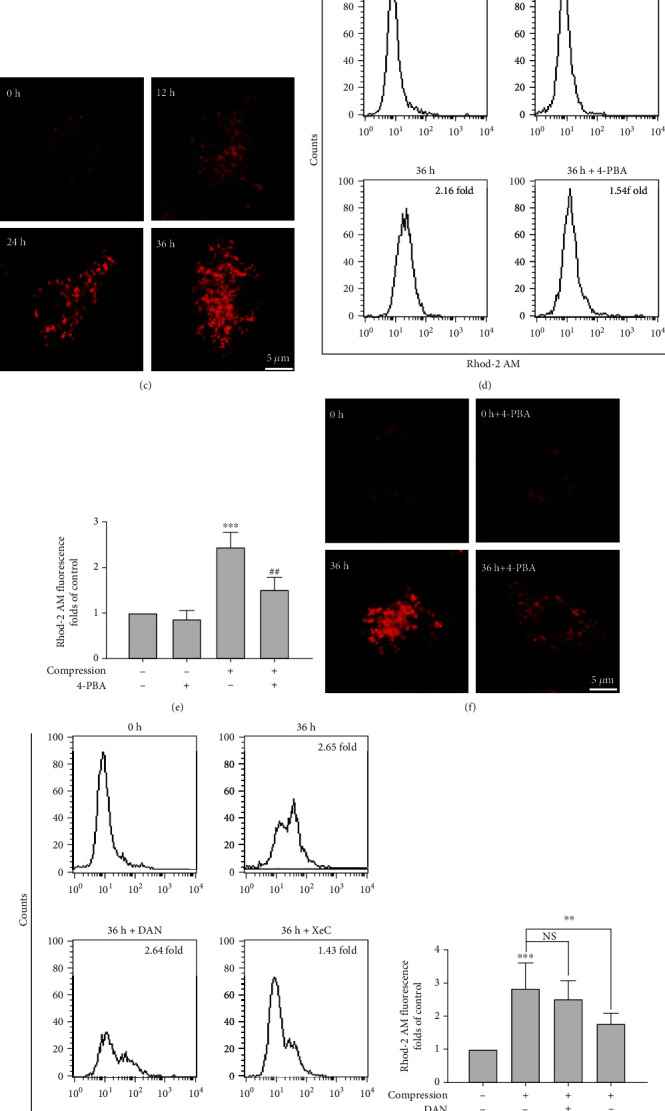
Ca^2+^ assembling in mitochondria may contribute to ERS-related Ca^2+^ release. (a, b) Representative histograms and statistical analysis of [Ca^2+^]_m_ detected by flow cytometry in compression-treated NP cells with Rhod-2 AM. NS means no significant difference. The values are expressed as the mean ± SD from three biological replicates (^∗∗^*P* < 0.01 and ^∗∗∗^*P* < 0.001 vs. control, ANOVA/LSD). (c) Typical fluorescence photomicrograph of in situ [Ca^2+^]_m_ staining with Rhod-2 AM. (d, e) Representative histograms and statistical analysis of [Ca^2+^]_m_ detected by flow cytometry in compression-treated NP cells with Rhod-2 AM. Cells were pretreated with 200 *μ*M 4-PBA for 1 h and then subjected to compression for 36 h. The values are expressed as the mean ± SD from three biological replicates (^∗∗∗^*P* < 0.001 vs. control, ^##^*P* < 0.01 vs. compression alone, ANOVA/LSD). (f) Typical fluorescence photomicrograph of in situ [Ca^2+^]_m_ staining with Rhod-2 AM. Cells were treated as in (d). (g, h) Representative histograms and statistical analysis of [Ca^2+^]_m_ detected by flow cytometry in compression-treated NP cells with Rhod-2 AM. Cells were pretreated with 50 *μ*M DAN or 2.5 *μ*M XeC for 1 h and then subjected to compression for 36 h. NS means no significant difference. The values are expressed as the mean ± SD from three biological replicates (^∗∗∗^*P* < 0.001 vs. control, ^##^*P* < 0.01 vs. compression alone, ANOVA/LSD). (i) Typical fluorescence photomicrograph of in situ [Ca^2+^]_m_ staining with Rhod-2 AM. Cells were treated as in (g).

We have revealed that ERS contributes to compression-induced programmed necrosis of NP cells. However, the underlying mechanisms remain unclear. Several studies have indicated that perturbation of ER Ca^2+^ homeostasis is involved in ERS and cell apoptosis [21]. Specifically, Ca^2+^ released from the ER translocates to the mitochondria to induce cell death in response to cellular stimuli [22]. Our flow cytometry results indicated that NP cells subjected to mechanical load showed significant upregulation of [Ca^2+^]_m_ at 24 and 36 h. The *P* values of each treatment group were 0.142, 0.002, and 0.0005, respectively (Figures 2(a)–2(c)). Interestingly, 4-PBA completely inhibited the upregulation of [Ca^2+^]_m_ in compression-treated NP cells (*P* < 0.01; Figures 2(d)–2(f)). However, the specific Ca^2+^ channels that determine compression-induced Ca^2+^ translocation have not yet been clarified. The ryanodine receptor (RyR) and IP_3_R are the main intracellular Ca^2+^ release channels located on the ER of most cell types. To further explain the role of the ER in Ca^2+^ redistribution, we employed Ca^2+^-channel inhibitors—xestospongin C (for IP_3_R) and dantrolene (for RyR)—to investigate the change in [Ca^2+^]_m_. As shown in Figures 2(g)–2(i), NP cells pretreated with xestospongin C exhibited a decrease in [Ca^2+^]_m_ levels compared with the compression group (*P* < 0.01). By contrast, dantrolene was unable to block the compression-mediated increase in [Ca^2+^]_m_ levels (*P* > 0.05). Our study indicates that compression induces ER Ca^2+^ efflux to the mitochondria via the IP_3_R channel.

### 3.3. The IP_3_R–GRP75–VDAC1 Complex Is Responsible for the Ca^2+^ Translocation between the ER and the Mitochondria

**Figure 3:**
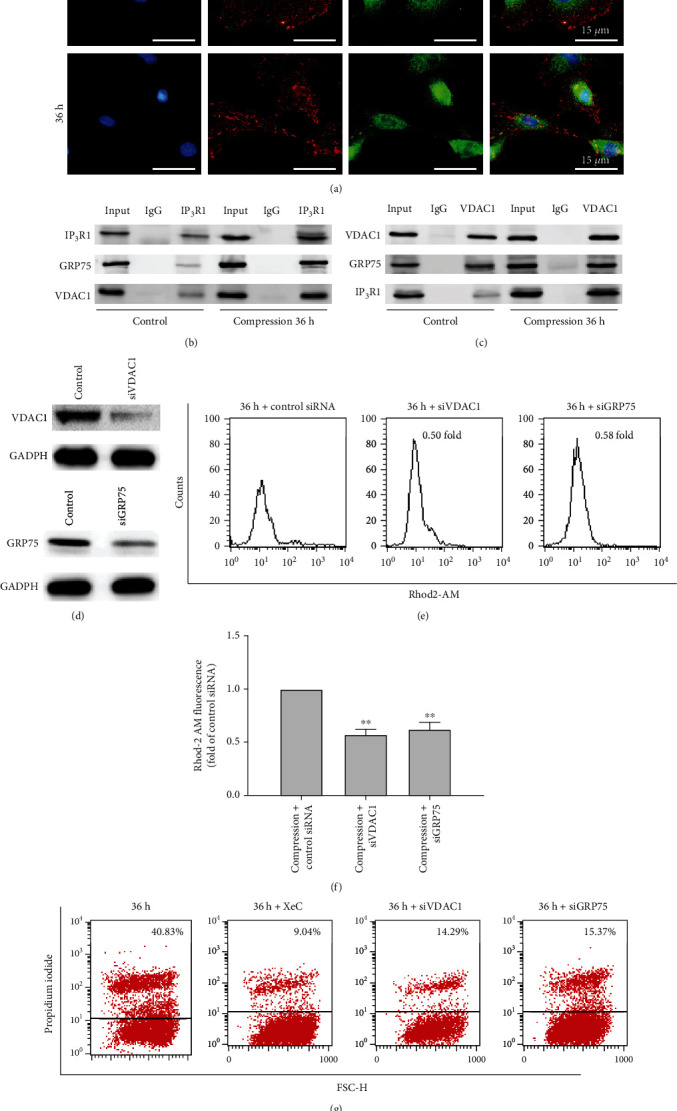
Compression-induced Ca^2+^ communication between the ER and the mitochondria relies on the IP_3_R–GRP75–VDAC1 complex. (a) Representative fluorescent images of the colocalization of IP_3_R1 and VDAC1 in NP cells treated with compression for 0, 12, 24, and 36 h. In the merged images, overlap (yellow), representing the colocalization of IP_3_R1 and VDAC1, was obviously increased after 36 h mechanical loading. (b, c) Representative western blot graphs of coimmunoprecipitations of IP_3_R1, GRP75, and VDAC1 in NP cells treated with compression for 36 h. (d) The typical western blot bands of VDAC1 and GRP75 in NP cells transfected with negative control siRNA (NC), VDAC1-siRNA, and GRP75-siRNA. (e, f) Representative histograms and statistical analysis of [Ca^2+^]_m_ detected by flow cytometry in compression-treated NP cells with Rhod-2 AM. NP cells were pretreated with VDAC1-siRNA or GRP75-siRNA and then exposed to 1 MPa compression for 36 h. The values are expressed as the mean ± SD from three biological replicates (^∗∗^*P* < 0.01 vs. control, ANOVA/LSD). (g, h) Representative dot plots and quantitative analysis of PI uptake in NP cells. NP cells were pretreated with 2.5 *μ*M XeC, VDAC1-siRNA, or GRP75-siRNA and then exposed to 1 MPa compression for 36 h. The values are expressed as the mean ± SD from three biological replicates (^∗∗∗^*P* < 0.001 vs. compression alone, ANOVA/LSD).

The interaction between ER and mitochondria induced by compression was evident (Figure 1(d), red arrows). This interaction may be responsible for compression-induced Ca^2+^ translocation. Previous studies have confirmed that the ER couples with the mitochondria and that an IP_3_R–GRP75–VDAC1 complex—essential for intracellular Ca^2+^ efflux into the mitochondria—is present at the ER-mitochondria interface [23]. As demonstrated above, IP_3_R is located in the ER and is responsible for compression-induced Ca^2+^ release from the ER pool. VDAC1 is a mitochondrial outer membrane protein, and GRP75 is a linker protein that tethers IP_3_R to VDAC1 to generate a molecular bridge for Ca^2+^ translocation [19]. To investigate the potential effect of the IP_3_R–GRP75–VDAC1 complex on Ca^2+^ accumulation in the mitochondria under excessive compression, we used confocal microscopy to analyze the colocalization of IP_3_R and VDAC1. The yellow areas represent colocalization between IP_3_R and VDAC1; these areas were more obvious under 36 h compression, demonstrating that compression enhanced IP_3_R-VDAC1 colocalization in NP cells (Figure 3(a)). Furthermore, the formation of the IP_3_R–GRP75–VDAC1 complex was confirmed by coimmunoprecipitation using IP_3_R or VDAC1 as bait. As shown in Figures 3(b) and 3(c), compared with the control group, the combination of IP_3_R, VDAC1, and GRP75 was enhanced when cells were subjected to mechanical loading. In summary, our studies demonstrated that the formation of the IP_3_R–GRP75–VDAC1 complex was significantly increased under continuous compression.

To confirm the impact of the IP_3_R–GRP75–VDAC1 complex on Ca^2+^ transmission from the ER pool to the mitochondria, we silenced GRP75 and VDAC1 expression by siRNAs in NP cells, and the levels of [Ca^2+^]_m_ were observed by Rhod-2 AM staining. As shown in Figures 3(d)–3(f), silencing of GRP75 and VDAC1 reduced the levels of [Ca^2+^]_m_ in NP cells exposed to mechanical load (*P* < 0.01). Moreover, the inhibition of IP_3_R by XeC and the silencing of either GRP75 or VDAC1 all reduced the percentage of PI-positive NP cells (*P* < 0.001; Figures 3(g) and 3(h)). These results indicate that the formation of the IP_3_R–GRP75–VDAC1 complex in NP cells under compression was responsible for the efflux of Ca^2+^ from the ER to the mitochondria and compression-induced NP cell death.

### 3.4. Inhibition of Compression-Induced Programmed Necrosis in [Ca^2+^]_m_-Protected NP Cells by Improving Mitochondrial Function and Inhibiting the PARP/AIF Pathway

**Figure 4:**
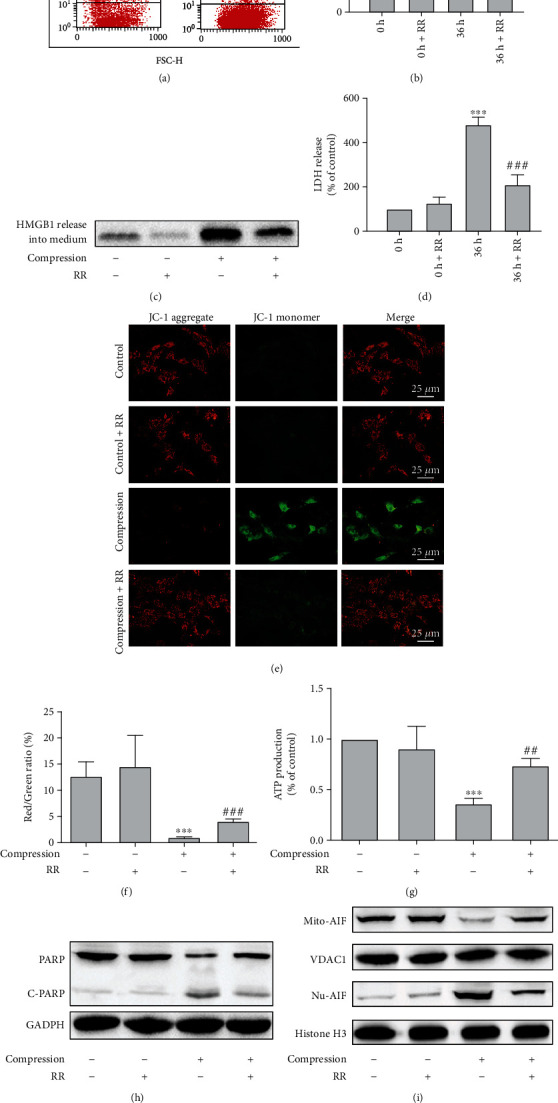
Ca^2+^ accumulation leads to mitochondria-related programmed cell death via the PARP/AIF pathway. (a) Representative flow cytometry images of PI uptake analysis for NP cells that were treated with 5 *μ*M RR for 1 h and then exposed to compression for 36 h. (b) Quantitative analysis of flow cytometry for cellular PI uptake. NP cells were treated as in (a). The values are expressed as the mean ± SD from three biological replicates (^∗∗∗^*P* < 0.001 vs. control, ^##^*P* < 0.01 vs. compression alone, ANOVA/LSD). (c) Representative western blot graphs of the levels of HMGB1 content in the media. NP cells were treated as in (a). (d) Histogram for statistical analysis of the LDH leakage in compression-treated NP cells. NP cells were treated as in (a). The values are expressed as the mean ± SD from three biological replicates (^∗∗∗^*P* < 0.001 vs. control, ^###^*P* < 0.001 vs. compression alone, ANOVA/LSD). (e, f) Typical fluorescence photomicrographs and quantitative analysis of mitochondrial membrane potential performed by a JC-1 probe. NP cells were treated as in (a). The values are expressed as the mean ± SD from three biological replicates (^∗∗∗^*P* < 0.001 vs. control, ^###^*P* < 0.001 vs. compression alone, ANOVA/LSD). (g) Quantitative analysis of ATP production in NP cells. NP cells were treated as in (a). The values are expressed as the mean ± SD from three biological replicates (^∗∗∗^*P* < 0.001 vs. control, ^##^*P* < 0.01 vs. compression alone, ANOVA/LSD). (h) Representative western blot graphs of PARP and C-PARP determined by WB. NP cells were treated as in (a). (i) Representative western blot graphs of mitochondrial and intranuclear AIF protein levels determined by WB. NP cells were treated as in (a). (j) Representative fluorescence images of the AIF staining in NP cells. NP cells were stained for nuclei by DAPI (blue) and for AIF by anti-AIF antibody (red). NP cells were treated as in (a).

We confirmed that compression induced ER-mitochondrial Ca^2+^ exchange in NP cells. To further explore the role of Ca^2+^ redistribution in IVDD, we preincubated NP cells with ruthenium red (RR), a mitochondrial Ca^2+^ uptake inhibitor. As seen in Figures 4(a) and 4(b), RR inhibited PI uptake in NP cells under compression (*P* < 0.01). Furthermore, the release of LDH and HMGB1 into the extracellular medium was inhibited by RR (*P* < 0.001; Figures 4(c) and 4(d)).

Mitochondrial dysfunction plays a critical role in IVDD progression. We first determined whether Ca^2+^ overload in mitochondria mediated the compression-induced NP cell necrosis by disturbing mitochondrial homeostasis. Mitochondrial inner membrane potential (*ΔΨ*_m_) is an essential indicator of mitochondrial function. JC-1 indicates mitochondrial polarization by shifting its fluorescence from green to red in a potential-sensitive manner. As shown in Figures 4(e) and 4(f), *ΔΨ*_m_ was significantly lower in compression-treated cells (*P* < 0.001); however, the reduction was greatly reduced when RR was present (*P* < 0.001). In addition, cellular ATP levels were significantly decreased after compression for 36 h (*P* < 0.001), whereas cotreatment with RR alleviated the compression-mediated ATP decrease (*P* < 0.01; Figure 4(g)). These results suggest that ER-mitochondrial Ca^2+^ exchange may mediate compression-induced programmed necrosis of NP cells by disrupting cellular bioenergetics.

PARP, located at the mitochondrial outer membrane, is an important protein that regulates energy metabolism and mitochondrial membrane potential [24]. Activated PARP leads to continued nicotinamide adenine dinucleotide (NAD^+^) depletion and loss of mitochondrial membrane potential, which then triggered AIF translocation from the mitochondria to the nucleus to cause chromatin condensation and DNA fragmentation, which leads to programmed necrosis of cells [25]. In this study, the expression of PARP was significantly decreased, whereas that of cleaved PARP (C-PARP) was elevated in NP cells exposed to compression (Figure 4(h)), indicating the activation of PARP. In addition, compression also induced a decrease in AIF in the mitochondria and increased AIF accumulation in the cell nucleus (Figure 4(i)). RR treatment decreased C-PARP expression and increased PARP expression (Figure 4(h)). Additionally, intranuclear accumulation of AIF was attenuated in the presence of RR (Figures 4(i) and 4(j)). Therefore, these results suggest that Ca^2+^ mediated programmed necrosis of NP cells under compression by facilitating the PARP-AIF pathway.

### 3.5. Increase in ROS Levels Contributes to Compression-Induced ERS, [Ca^2+^]_m_ Overload, and Programmed Necrosis

**Figure 5:**
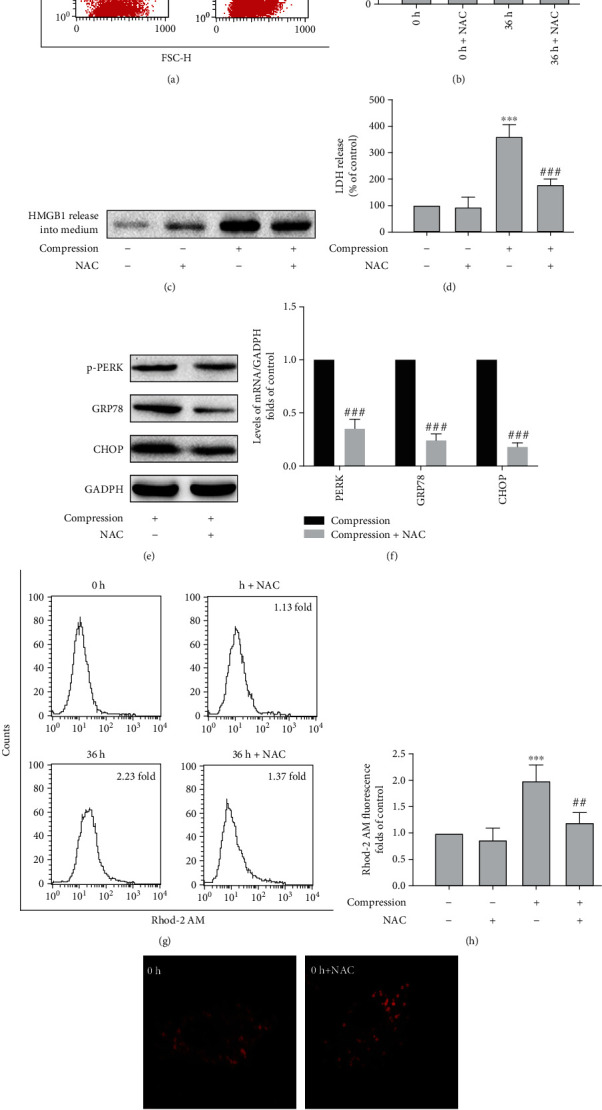
Increased ROS levels relate to compression-induced ERS, [Ca^2+^]_m_ overload, and programmed NP cell death. (a) Representative flow cytometry images of PI uptake analysis for NP cells that were treated with 5 mM NAC for 1 h and then exposed to compression for 36 h. (b) Quantitative analysis of flow cytometry for cellular PI uptake. NP cells were treated as described in (a). The values are expressed as the mean ± SD from three biological replicates (^∗∗∗^*P* < 0.001 vs. control, ^###^*P* < 0.001 vs. compression alone, ANOVA/LSD). (c) Representative western blot graphs of the levels of HMGB1 content in the media. NP cells were treated as shown in (a). (d) Histogram for statistical analysis of the LDH leakage in compression-treated NP cells. NP cells were treated as in (a). The values are expressed as the mean ± SD from three biological replicates (^∗∗∗^*P* < 0.001 vs. control, ^###^*P* < 0.001 vs. compression alone, ANOVA/LSD). (e) Representative western blot graph of the levels of p-PERK, GRP78, and CHOP protein expression in NP cells exposed to compression. NP cells were treated as in (a). (f) The mRNA levels of PERK, GRP78, and CHOP detected by RT-qPCR in NP cells under compression conditions. NP cells were treated as in (a). The values are expressed as the mean ± SD from three biological replicates (^###^*P* < 0.001 vs. compression alone, Student's *t*-test). (g, h) Representative histograms and statistical analysis of [Ca^2+^]_m_ in compression-treated NP cells detected by flow cytometry with Rhod-2 AM. NP cells were treated as in (a). The values are expressed as the mean ± SD from three biological replicates (^∗∗∗^*P* < 0.001 vs. control, ^##^*P* < 0.01 vs. compression alone, ANOVA/LSD). (i) Typical fluorescence photomicrograph of in situ [Ca^2+^]_m_ staining with Rhod-2 AM under a fluorescence microscope. NP cells were treated as in (a).

It has been reported that ROS are crucial mediators in the pathogenesis of IVDD [26, 27]. To assess ROS accumulation and related damage in NP cells subjected to compression, total ROS was detected using the fluorescent dye H_2_DCFH-DA, and a quantification of oxidative DNA damage—8-hydroxydeoxyguanosine (8-OHdG) quantitative analysis—was carried out to evaluate ROS damage in NP cells. The results indicated that compression caused ROS accumulation in NP cells and increased levels of 8-OHdG in rat NP cells, suggesting that oxidative damage occurs in NP cells subjected to compression (Figure S3). We then applied NAC to scavenge ROS and found that compression-induced programmed necrosis was significantly attenuated by NAC when compared with the compression group in the absence of NAC, as detected by PI flow cytometry (*P* < 0.001), PI staining, and cellular release of HMGB1 and LDH (*P* < 0.001; Figures 5(a)–5(d)). Interestingly, NAC also efficiently inhibited the upregulation of ERS markers in NP cells exposed to compression (Figures 5(e) and 5(f)). Therefore, compression-induced ROS may serve as an activator of increased ERS, resulting in programmed cellular necrosis. NAC also inhibited compression-induced [Ca^2+^]_m_ overload (*P* < 0.01; Figures 5(g)–5(i)). In summary, our results indicate that compression induced ROS accumulation that excited ERS-related [Ca^2+^]_m_ overload and led to programmed necrosis.

## 4. Discussion

In the present study, we first demonstrated that radical ERS contributed to programmed necrosis of NP cells under compression. Under mechanical load, Ca^2+^ was released from the ER and translocated to the mitochondria via the IP_3_R–GRP75–VDAC1 complex, which resulted in programmed necrosis through the PARP/AIF pathway. Our findings revealing the molecular basis of programmed necrosis of NP cells induced by compression have tremendous potential for the treatment of IVDD.

The ERS response system has dual functions in the execution of cell death. Appropriate ERS protects cells against the death response to a harmful stimulus. However, aggravated ERS promotes cell apoptosis by initiating a proapoptotic mechanism [28]. Previous studies have confirmed that ERS plays a vital role in IVDD pathogenesis. IVDD was alleviated to a certain degree by targeting ERS induced by oxidative stress or advanced glycation end products (AGEs) [12, 29]. Nevertheless, whether ERS accelerates NP cell programmed necrosis under compression has not yet been elucidated. Mechanical loading is consistent and inevitable stress on human discs. Compression may be of greater clinical relevance than chemicals [30]. In this study, we observed increased expression of the ERS-related proteins GRP78, CHOP, and p-PERK, along with significant ER deformation in response to mechanical loading. Pretreatment with the chemical ERS inhibitor 4-PBA significantly decreased compression-induced NP cell death. Our study is the first to indicate that ERS contributes to compression-induced NP cell programmed necrosis.

The ER is an important organelle that regulates intracellular Ca^2+^ levels [21]. In response to pharmacological and physiological stimuli that disrupt ER homeostasis, Ca^2+^ is released by the ER through two ER-specific Ca^2+^ channels: RyR and IP_3_R. Because of close physical contact with the ER, the mitochondria acquire a remarkable Ca^2+^ fraction [21]. Our results demonstrated that compression induced Ca^2+^ elevation in the mitochondria of NP cells; this was attenuated by XeC (IP_3_R inhibitor) but not by dantrolene (RyR inhibitor). Interestingly, RyR and IP_3_R were both found to be responsible for increased Ca^2+^ release in NP cells induced by AGEs [31]. AGEs have been reported to crosslink in the annulus fibrosus and lead to increased compressive stiffness, torque range, and failure torque [32], whereas excessive mechanical loading leads to extracellular matrix remodeling and a decreased number of intervertebral disc cells [33]. These pathogenic differences between AGEs and compression may lead to different roles of RyR in regulating Ca^2+^ redistribution in NP cells, while further studies may be required to further clarify the roles of RyR.

Increasing data have verified that Ca^2+^ translocation from the ER to the mitochondria by the IP_3_R–GRP75–VDAC1 complex results in Ca^2+^ overload in the mitochondria, which leads to the opening of mitochondrial permeability transition pores, release of cytochrome c, and activation of caspases to induce cell apoptosis [19, 34]. Our results indicate that IP_3_R strikingly interacts with GRP75 and VDAC1 in NP cells under mechanical load conditions, providing the molecular basis for Ca^2+^ translocation. To explore the potential impacts of a reinforced IP_3_R–GRP75–VDAC1 complex in [Ca^2+^]_m_ excess and NP cell death, we inhibited different proteins in the axis for further exploration. The current results clearly demonstrated that inhibition of IP_3_R, GRP75, or VDAC1 prevented compression-induced [Ca^2+^]_m_ overload and decreased NP cell programmed necrosis. Our results suggest that the IP_3_R–GRP75–VDAC1 complex facilitates NP cell programmed necrosis by stimulating Ca^2+^ efflux from the ER to the mitochondria. The mechanism by which compression induces IP_3_R–GRP75–VDAC1 complex assembly on the ER requires further investigation.

Ca^2+^ overload in the mitochondria usually serves as intracellular signaling for PARP activation [35, 36]. PARP activation leads to PARP-mediated mitochondrial dysfunction, as demonstrated by mitochondrial membrane depolarization, overproduction of superoxide, and loss of cardiolipin content [37]. Besides, in response to damage stimulation, the DNA repair enzyme, PARP, results in the massive synthesis of poly(ADP-ribose) (PAR) from nicotinamide adenine dinucleotide (NAD+), leading to rapid exhaustion of intracellular NAD+ and ATP. Afterwards, AIF escapes from the mitochondria to the nucleus and then combines with DNA and RNA to bring about chromatolysis and cell programmed necrosis [38]. Supportively, we previously reported that the PARP was activated in H_2_O_2_-treated disc cells to exert cell necrosis [39]. Thus, PARP/AIF may be critical in Ca^2+^ overload-induced mitochondrial dysfunction and cell death in NP cells under prolonged compression. In our study, we found that Ca^2+^ accumulation in the mitochondria induced by compression significantly impaired mitochondrial membrane potential and ATP synthesis. Also, compression increased PARP hyperactivation and the translocation of AIF from the mitochondria to the nucleus, which could be inhibited by RR. Therefore, Ca^2+^ efflux to the mitochondria from the ER mediated compression-induced NP cell programmed necrosis via activation of the PARP/AIF pathway.

Excessive accumulation of ROS, including NO, peroxynitrite, and nitrotyrosine, has been identified in degenerative discs [40]. Multiple microenvironmental factors in discs, such as mechanical loading and proinflammatory cytokines, contribute to increased ROS levels in disc cells [40–42]. Importantly, excessive ROS accumulation may disrupt protein synthesis and conformation, leading to protein deformation and unfolded protein response [43]. Inhibition of ROS significantly reduced ERS activation and ERS-related Leydig cell apoptosis in rats [44]. These findings were consistent with those of our study in that ERS was downregulated after NAC administration and compression-induced programmed necrosis of NP cells was decreased. Although mitochondrial dysfunction can be caused by either Ca^2+^ overload or ROS production [45], it is unclear whether Ca^2+^ overload in the mitochondria is regulated by ROS production. Our findings suggest that NAC largely decreases the accumulation of compression-induced Ca^2+^ in the mitochondria. Considering NAC downregulated ERS, which was essential in compression-induced [Ca^2+^]_m_ overload, compression-induced excessive ROS may increase Ca^2+^ accumulation in the mitochondria via radical ERS.

## 5. Conclusions

Our results indicate for the first time that compression-induced ERS plays a crucial role in mediating NP cell programmed necrosis. Under mechanical loading, Ca^2+^ is released from the ER and translocated to the mitochondria through the IP_3_R–GRP75–VDAC1 complex. The upregulation of mitochondrial Ca^2+^ perturbs mitochondrial function and activates the PARP/AIF pathway to induce programmed necrosis. Moreover, excessive ROS accumulation is a critical activator of ERS, leading to mitochondrial Ca^2+^ accumulation and consequently programmed necrosis. These findings may provide new insights into the molecular mechanisms underlying IVDD and have important clinical implications for IVDD treatment. However, further studies involving humans and in vivo experiments are needed to verify our results.

## Data Availability

The data used to support the findings of this study are available from the corresponding authors upon request.

## Supplementary Materials

Supplementary Materials Figure S1: ERS makers are upregulated in degenerative discs. (a) Quantification of GRP75-positive and CHOP-positive cells in normal and degenerated discs detected by immunohistochemistry. The values are expressed as the mean ± SD from three independent experiments (^∗∗∗^*P* < 0.001 vs. normal group, Student's *t*-test). (b) Representative fluorescent images of GRP75 and CHOP staining in normal and degenerated discs detected by immunofluorescence. (c) Quantification of GRP75-positive and CHOP-positive cells in normal and degenerated discs detected by immunofluorescence. The values are expressed as the mean ± SD from three independent experiments (^∗∗∗^*P* < 0.001 vs. normal group, Student's *t*-test). Figure S2: 4-PBA protects NP cells against compression-induced necrosis. (a) Representative western blot graphs of the levels of p-PERK, GRP78, and CHOP in NP cells. Cells were pretreated with 200 *μ*M 4-PBA for 1 h and then subjected to compression for 36 h. (b) Typical fluorescence photomicrograph of in situ PI staining in NP cells. Cells were treated as in (a). Figure S3: compression leads to increased ROS levels. (a) Representative fluorescent images of the levels of ROS in NP cells exposed to compression for 0, 12, 24, and 36 h (scale bars = 100 *μ*m). (b, c) Representative histograms and statistical analysis of ROS in compression-treated NP cells detected by flow cytometry with H_2_-DCFHDA. Cells were treated as in (a). The values are expressed as the mean ± SD from three independent experiments (^∗^*P* < 0.05, ^∗∗^*P* < 0.01, and ^∗∗∗^*P* < 0.001 vs. control, ANOVA/LSD). (d) Histogram for statistical analysis of the content of 8-OHdG in compression-treated NP cells detected by ELISA. Cells were treated as in (a). NS means no significant difference. The values are expressed as the mean ± SD from three independent experiments (^∗∗∗^*P* < 0.001 vs. control, ANOVA/LSD).
