# Supplementary material for: Reactive Oxygen Species Regulate Endoplasmic Reticulum Stress and ER-Mitochondrial Ca2+ Crosstalk to Promote Programmed Necrosis of Rat Nucleus Pulposus Cells under Compression
Source: Oxid Med Cell Longev. 2021 Mar 16;2021:8810698. doi: 10.1155/2021/8810698 (PMC7987452; doi:10.1155/2021/8810698)
Supplement: Supplementary Materials — Figure S1: ERS makers are upregulated in degenerative discs. (a) Quantification of GRP75-positive and CHOP-positive cells in normal and degenerated discs detected by immunohistochemistry. The values are expressed as the mean ± SD from three independent experiments (∗∗∗P < 0.001 vs. normal group, Student's t-test). (b) Representative fluorescent images of GRP75 and CHOP staining in normal and degenerated discs detected by immunofluorescence. (c) Quantification of GRP75-positive and CHOP-positive cells in normal and degenerated discs detected by immunofluorescence. The values are expressed as the mean ± SD from three independent experiments (∗∗∗P < 0.001 vs. normal group, Student's t-test). Figure S2: 4-PBA protects NP cells against compression-induced necrosis. (a) Representative western blot graphs of the levels of p-PERK, GRP78, and CHOP in NP cells. Cells were pretreated with 200 μM 4-PBA for 1 h and then subjected to compression for 36 h. (b) Typical fluorescence photomicrograph of in situ PI staining in NP cells. Cells were treated as in (a). Figure S3: compression leads to increased ROS levels. (a) Representative fluorescent images of the levels of ROS in NP cells exposed to compression for 0, 12, 24, and 36 h (scale bars = 100 μm). (b, c) Representative histograms and statistical analysis of ROS in compression-treated NP cells detected by flow cytometry with H2-DCFHDA. Cells were treated as in (a). The values are expressed as the mean ± SD from three independent experiments (∗P < 0.05, ∗∗P < 0.01, and ∗∗∗P < 0.001 vs. control, ANOVA/LSD). (d) Histogram for statistical analysis of the content of 8-OHdG in compression-treated NP cells detected by ELISA. Cells were treated as in (a). NS means no significant difference. The values are expressed as the mean ± SD from three independent experiments (∗∗∗P < 0.001 vs. control, ANOVA/LSD). [file 8810698.f1.docx]

**supplementary material**


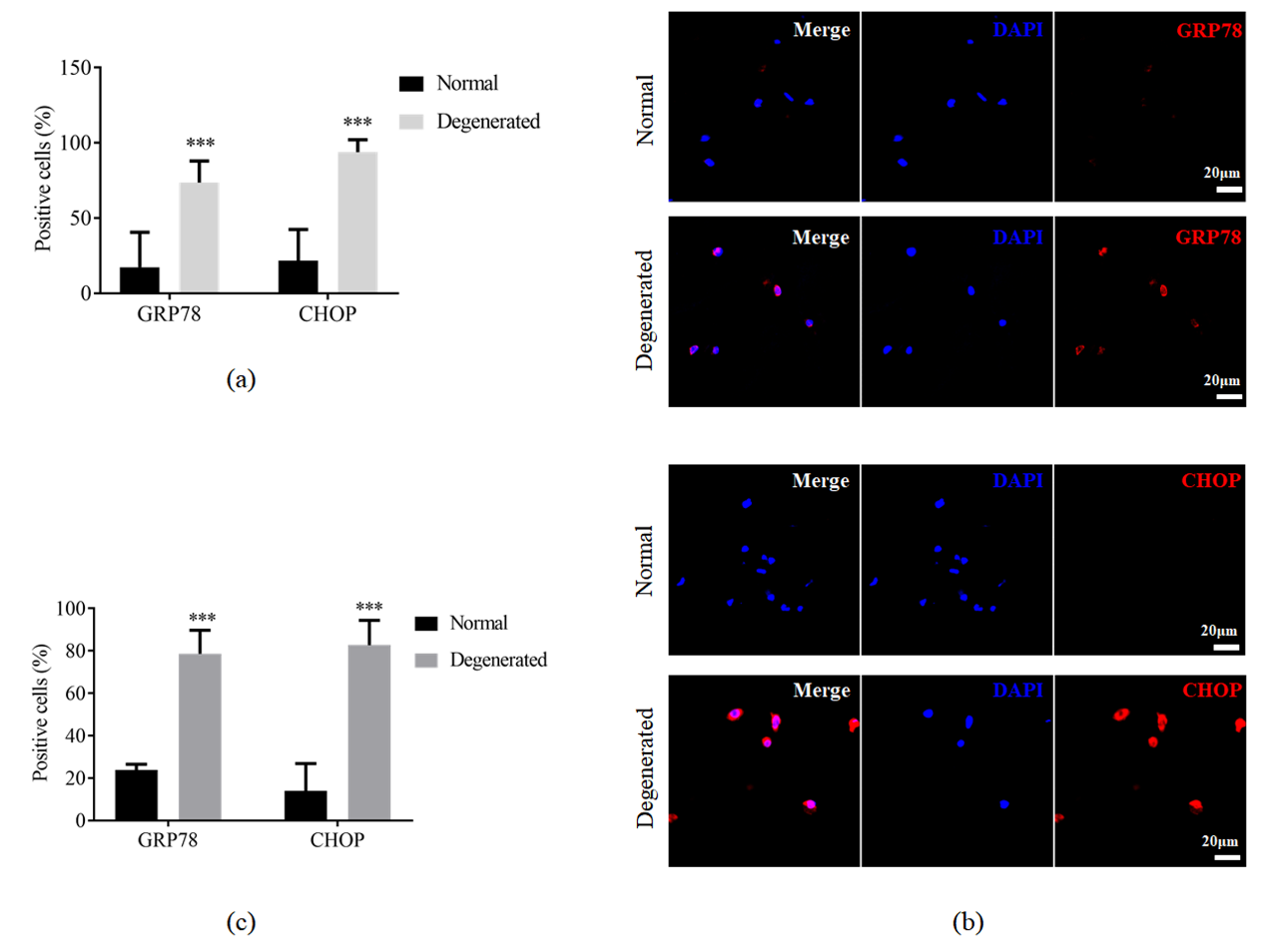


Figure S1: ERS makers are upregulated in degenerative discs. (a) Quantification of GRP75-positive and CHOP-positive cells in normal and degenerated discs detected by immunohistochemistry. The values are expressed as mean ± SD from three independent experiments (^***^*P*<0.001 vs. Normal group, Student's *t* test). (b) Representative fluorescent images of GRP75 and CHOP staining in normal and degenerated discs detected by immunofluorescence. (c) Quantification of GRP75-positive and CHOP-positive cells in normal and degenerated discs detected by immunofluorescence. The values are expressed as mean ± SD from three independent experiments (^***^*P*<0.001 vs. Normal group, Student's *t* test).


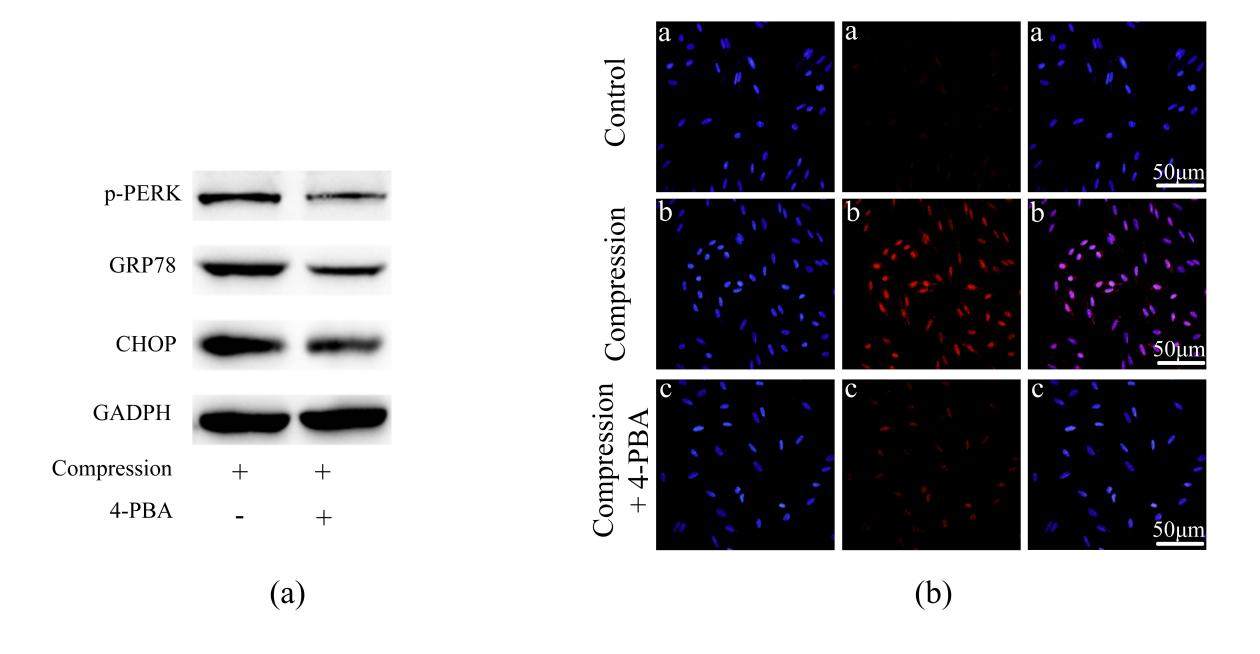


Figure S2: 4-PBA protects NP cells against compression-induced necrosis. (a) Representative western blot graphs of the levels of p-PERK, GRP78 and CHOP in NP cells. Cells were pretreated with 200 μM 4-PBA for 1 h and then subjected to compression for 36 h. (b) Typical fluorescence photomicrograph of in situ PI staining in NP cells. Cells were treated as in (a).


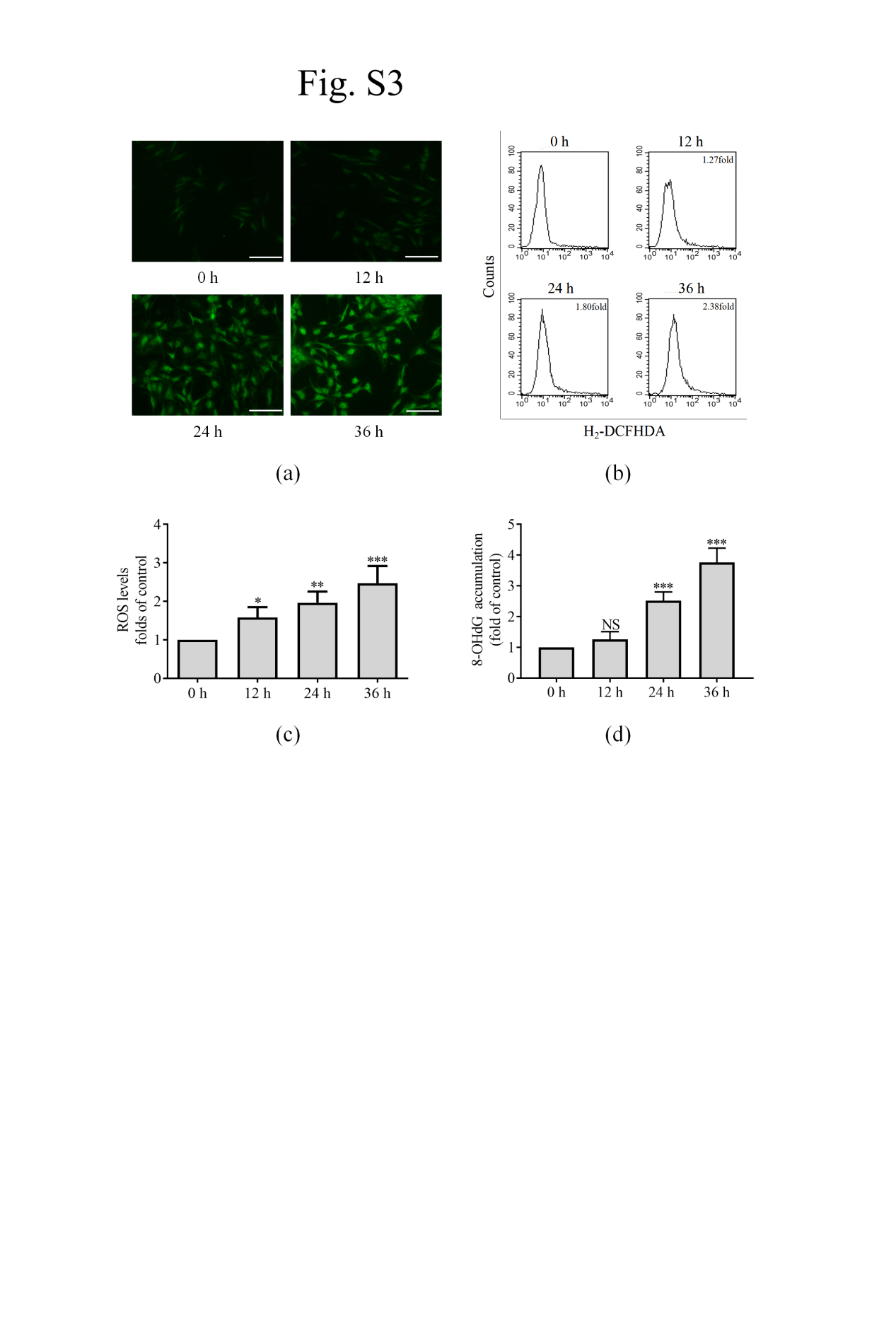


Figure S3: Compression leads to increased ROS levels. (a) Representative fluorescent images of the levels of ROS in NP cells exposed to compression for 0, 12, 24 and 36 h. (Scale bars = 100 μm). (b,c) Representative histograms and statistical analysis of ROS in compression-treated NP cells detected by flow cytometry with H_2_-DCFHDA. Cells were treated as in (a). The values are expressed as mean ± SD from three independent experiments (^*^*P*<0.05, ^**^*P*<0.01, ^***^*P*<0.001 vs. control, ANOVA/LSD). (d) Histogram for statistical analysis of the content of 8-OHdG in compression-treated NP cells detected by ELISA. Cells were treated as in (a). NS means no significant difference. The values are expressed as mean ± SD from three independent experiments (^***^*P*<0.001 vs. control, ANOVA/LSD).
